# Supplementary material for: Detection of Fused Genes in Eukaryotic Genomes using Gene deFuser: Analysis of the Tetrahymena thermophila genome
Source: BMC Bioinformatics. 2011 Jul 11;12:279. doi: 10.1186/1471-2105-12-279 (PMC3143110; doi:10.1186/1471-2105-12-279)
Supplement: Additional file 1 — Results of Gene deFuser for the Tetrahymena thermophila genome. This zip file contains the raw results of the analysis of the Tetrahymena genome using Gene deFuser. To view the contents, unzip the file and open the Final_Tet.html file in the resulting folder. [file 1471-2105-12-279-S1.ZIP › Results/109.m00143.html]

Gene deFuser -- Results of Job Final\_Tet

 


Gene deFuser

| Home | Retrieve Results | References | Help |
| --- | --- | --- | --- |

Back to Main Results of Job Final\_Tet

# Query Name: 109.m00143

Candidate fusion gene

## Query Sequence:

MDDKFEAQQQLNRLQENQYQSYQDRVNMFQQNNKILEQVRSHFKHPEQYEGDHLGGDLAWQNEYYKLYNDRLQKQFEDLLTGKGFSVYQKDLIIKPVSGNQFEELLISSDRLSPLLKALEDENKILKEEVSHLSGDLKRFIGMVELLLEENKYIREHINQKNRDITKLIETIGLNDGEEINDLKHKIHILTEENNILIKHLDEMKRFREEYDNFVYENDQEINRAREVIEKTKDDLEEALKREESLTKQLAQIEPKFTKYSQKVAELEEQVEELQNKLNHAKNDNQTLKSEYLHYKRQLAFKVQMINYILLINQSHYEDLSRKRGDENDQLLQENNMLMNEKKEFKQQLLFKDRQIEELKQQVLSFRKDVETKGYDLDELSQAVNEFKNQIQVEKQRAESALQRERQMREQFEIIQSESQKNQIKCEQLERQYNSLLESSRQELQDKHIKYEGLIEKQKIKNREEMDSKDEEITDLKTKIVEKDRIIQQLNNELERRNSDVNDFKKKLQDLKESRNLLDRQAQELIQLKNENRDLHNNLLELKNSKASFSGGEKEYKQMLNQIREFEISISQLEKNNTLLKEENDHLKMQVQDQKQEVREFQEKLALSKKEIQQLKDERDEMMQKQMEEVSFFILIKPVYLAKFQIIFNKITRESIKWVGMIKALNQDRINLILTAVKVEMQVHWIINLICNTNEPNQPTKINQIKIKTKIKIDINIIINQLIIKQDQFVQCNYNRRKLFQLSSKKEAKQNKNIGMSKNILRIREDWEKRIQTFQQSELHQERKSNIRYDLIKIFVSDRSIELESKYFPIVNAITQENDKYDFLLTLLLLIADKLDEYLNSKDQKSSKILINIIIHALQKAPFVKKKQEALDIVILALEIVVKINLYTLKNPQQQQVDLLESDDEISENKKQNSQQQNSIIGNQIVEEQNQEKQKNEDHEFFLQNDLAVEIEENISNLITKAHIELLKQFELMESVDKISAQKLYYYLNFVLLYSPFNEQLFTQINKLYLLFYENFCQICNSFLIKFEESFYYALDILYNAPINALFLSLKGINKEGNPGYSKDLNEQLIQEVTSILTEVMVILPQQKQEDMLKQVISFLRIAKQKDDFLFNETINIFNKLKSQFININTNLSISLMCAIAENDDTFILNPSNQVWKLEEINEYAIIIKDKSERINANSSDLTFLLRLFYFYTKVYEYLNKENELIIIKSYEQTIFNKSASIFSYEKIAHLILTESFSDSKKRLFFMIVGIMVMTFRERKECRMTIQNVIIKLLNKKQDNYENQFEMVKYSFLCNMITNMYQKISQIQIYNAQKQYKLVQFYQYDSPLQFTMVFDKDLVQFDLFIQNIYEKLCVIATVNNDPLLLKEILCLTQSLQNKGESFALYSGAFKKMFLKLMPVVTPFHLNQILIFSTRGEYFPIYEKLFQKQVLGQNLLIDDDYQLINKTFDEVYFDKMSFIQINESNFLDVYIKYLEKVQDNQIKKYFLNVVYALAKNSFENTIFLSKPKVFQNIVSCLAKEQNQENRKLFLNLAQVILSEGVSAHHVKSITQQILNSQQDKYFCMLHESIRLLEDIINEQNSPSHITPYYYFKGRDSILMLDEKIPFVDNCSKGFSVLACLKLESVANFYDSSIMIKDVQNILYKANTSQDSSLPSTADSLKKNKSSDNFDPQPKQCLFCFVSKDDKKLVVYIIPGQSNKKAILRICFIQDSISIDIDFDYGNFSECSKWHILQINYSEKNGSEGFTVAFNGIVKQPISNQNRRYGKYVDNFKVLKQFSIGAEISPAKNCDNFVIEGSFFNGFINQLYIFNESIQIERTKIKLQEYLTEMYYNTNLREKIILKLNTEKNTLSKQNEGDQNLYSSLDRQNSKQNCDRQCKYHIQYGDNRDENIMLEAPICLTFLKQKRKLIKQSFLNMFKSDDISVKTEFPHYKIVSRGIRFLEFSHFLEALLSIGNIEIFIYILDLIIGNKELNSEQKMTIASAVFQLMSVMIQQDQEIIMYFDQNSKGMHTLKMLIKAIVKENKGCSIQFLESFKQFIEIGFPYQECNSTDFFDDQADMTWYARQVALQEIFFCYQIWGQCSFKIQRSVYVFILSFLEMDNETFDTVFRIVQNESLVPLFEVLQNNFNEKQIRTENNENDCKELRDLKGYLLKIINNIYTSAFIKNEVQLKRQDYKSLIELLLKNICYAEPGQLQLDLVRILQKIFNAKTPVEVISIIQNKEIESLEQIVVAILIHTCTMKQGSQQNREYFYDSDFSQQVNHFDELISISVQLLLEYYLKSVKKFDSSSIQIIQKQIQNLDSILPQYLHKETITILIEYLNKSLIEKWRIEKTIMEILFTRVPHLEGTQQIMVIKDIKKVLGILYNRHINALNKDQIDEDFKQFLHKLVINPQFIQIVENILYINFEDEESLKIVQDSSQEILDYIINFLIWGEIDGAKLLQLTQVNSKDSSQILLATQKYNLIELFFYVENIVYFYPNLISNQEAIYQKVIHSYIEFFGNNILCLENSSPQIKKELEGQIDLNATFNYLEKIVMHKLVLTNGGVLRSVIDTIFVSYRNSTDIGVKNGYLQDLNIFLMRDVGNSQQKEQKLFTESETINLIEYRNEFDRNIFTEYFKDYILTFALQEYFVTTNKTLKDSLYKLIYNSYYDKMQRSWSSFDTMQIGNYQYNLQISPYENLQQFLDIFNNKIEVKIDNNNLIEEIQDFFVEVKELRNFPNRNHEKLKDILESHKNLFLDQIIPIIDNYIGVQVQYVRHINGIFYKLREQVFQEAKREKINIFYSIHSQSASTKLKQQQIIDTYKQLCEDSFQKYYQLENEQLIETAKVWKSKKKRLVSERGLWFEENSSEKIKWKINKVEDRLRRKFFLKRDKKWLDKKFFTDPKYQKQQILPSKQQQEENDSSKKQFNLLGSFENETDTQDLNQNLAIQKDQSLIEDKQPTIRQVQSTLIEDPASIKRNQSSKQLEKDSSQQPMSPQKNEEVIIEQFDFKNNVQSASSKIRSSSFNNSQKIVENNMIDLICEAEQVTQTGVVYGILSISKHFQNQFLSQMKFLKDQKGAIIFSSKDQRIKPNIFGCIDHQLIQEQKTKVINVKNVKLAISRRYLFQEIAFELFTDDQKVIFFNVYKPQIKENFLSIIKRMNENCNVVINRKLEFQKSGIKDDWKKGRITNFDYLMQLNTYSGRSFNDLSQYPVFPWIIRDYKSSELKLEEDSFYRPLNQPIGAINRNRFLDHFYERFQHSSPKDRDTYFMYGTHYSNATIVLTLLMRMEPFAGLHFTQQSNKFDHADRLFHSIHDQWESGQLNSADVKELIPEFYYLPDFLYNVNELFLGKKQDNTEVDRVILPEWIKRSTDTPEEFVFRMRQALESPFVSKNLHSWIDLIFGSKQIGQKAIDNENLFHPSSYAENFNIKGLDEAQILAEKAKVIYFGQTPFRLFDEDQKPRDDSGLITMPKIKEFEFYNKITPKYQKVCQESSLEYVTANNRYVGLMERDLNKFYFNIFKCKTSILEKDFTYRRGKIEIKKSPEISFRNSVLLYRTFILSVAHGDKNIIVKNFKDEKYEAQINSLHKKIITCMDVCEEFIVCGSRDCRLSVYSVGVKQNILKNFLRKEDIEIKHLRMIYGHHNEISAVKLDQTLLIIVSIDMDGLGLIHELKTMRFLHSFKLPDLQDSECVQFLEIHQCGFILIVTNQKRLFIFTMNGNLYSECSFSYISNQNEEKITDVKFLSSLSSFIYLSTNKGVIYYLDLFEIKAKQIQNMTPAFSKFQPNSEHITSIYFHYQVYCILKEMEISVSFKKKTKLETLQCSQSKWEQKNTQISCLRIEKNTLIIKFNKKLLSYDFKNYRPKNTPTNQDTDHNRDRQTDKYILEKHVQSWYDQGQGPWQDQLASTIFFTSFQLMICQFDQINRQKNNQLNKAKKKKVDLMDQMSQNQQQHEMAYYKCKAINQNSFKCKQHQSLFILKTPKVSEINEPTLSKIQNIVYQEMKRELSQKGYDEFRFLGQGAQGLVVLAKEKSTNKRYAIKGIHIKDSKDNVDQVILGQVQQEIKMLNQCKGSPYVVNLLHQIEGNDFIFLVLTECLGTLNEIIKNTQNERLNELSAIKYAKDIAEGLYFIHSKKCLVNDLKMDNILIDYNGNAVVSDFGLADNLPQQSGYAIEEYVKFFLHLLKKKYDAYLFILIQLKKGNIICQAPECYDSSKFDIYSNEYIKLGIAVKKQPSQRVFIDKSLQLLLKCYLIYTMISGFQANLVFSKHQPLQYNADFQNFNYEKQLRYIIDGLTNFIPRNRLKVKEVLIILKGIISFEFNLDSKFIEVIDDAAAQQHISQFTQDIEYVQGFENKFYPIIKNEKELFQYLNSQIDAQKEKHVFQKQPELGHQDQIQKQNTEKKQDVQNYQQNQEQIKIDQLDNNQPTQQFIYIHQLLCMKNKKLLDFYD

### Significant Ortholog Group Hits and their Scores:

| N terminus | | C terminus | |
| --- | --- | --- | --- |
| [S] KOG4674 Uncharacterized conserved coiled-coil protein | 59.2657419804232 | [TU] KOG1786 Lysosomal trafficking regulator LYST and related BEACH and WD40 repeat proteins | 23.1578947368421 |
| [Z] KOG0161 Myosin class II heavy chain | 41.9862779134533 | [U] KOG1787 Kinase A-anchor protein Neurobeachin and related BEACH and WD40 repeat proteins | 21.7524357621548 |
| [U] KOG0946 ER-Golgi vesicle-tethering protein p115 | 26.6666666666667 | [T] KOG0696 Serine/threonine protein kinase | 15.6421838000639 |
| [ZR] KOG4568 Cytoskeleton-associated protein and related proteins | 13.3547522286284 | [T] KOG0695 Serine/threonine protein kinase | 14.9198026918925 |
| [S] KOG4643 Uncharacterized coiled-coil protein | 12.5963098892901 | [T] KOG0690 Serine/threonine protein kinase | 12.2162762524322 |
| [T] KOG0612 Rho-associated, coiled-coil containing protein kinase | 11.6663549081249 | [T] KOG0986 G protein-coupled receptor kinase | 10.609549263163 |
| [Z] KOG0247 Kinesin-like protein | 9.44601650229338 | [T] KOG0694 Serine/threonine protein kinase | 6.89124812843351 |
| [K] KOG4673 Transcription factor TMF, TATA element modulatory factor | 8.5171913134697 | [R] KOG0611 Predicted serine/threonine protein kinase | 6.15882218990855 |
| [T] KOG0976 Rho/Rac1-interacting serine/threonine kinase Citron | 8.18695413077821 | [T] KOG0574 STE20-like serine/threonine kinase MST | 5.16644319966984 |
| [S] KOG4415 Uncharacterized conserved protein | 7.84555882365536 |  |  |
| [K] KOG0963 Transcription factor/CCAAT displacement protein CDP1 | 6.6330984854143 |  |  |
| [UR] KOG4677 Golgi integral membrane protein | 6.15882218990855 |  |  |
| [L] KOG0962 DNA repair protein RAD50, ABC-type ATPase/SMC superfamily | 5.98619912385187 |  |  |

#### Graphs (click to enlarge):

|  |  |
| --- | --- |
| BLAST of Query Sequence | Location of Ortholog Group Hits |
|  |  |

Contact: Andre Cavalcanti\_\_\_\_\_Last Modified September 14, 2010
